# Opening the black box: study of the multi-target anticancer mechanisms of *Ecballium elaterium* cucurbitacins *via in vitro*, network pharmacology, and molecular docking analyses

**DOI:** 10.1039/d6ra07915f

**Published:** 2026-10-06

**Authors:** Enver Saka, Orchid A. Mahmoud, Omayma A. Eldahshan, Abdel Nasser B. Singab, Ismail Koyuncu, Gokhan Zengin, Sakina Yagi, Meng-Yao Li

**Affiliations:** a Department of Biology, Faculty of Science, Selcuk University Konya Türkiye; b Department of Pharmacognosy, Faculty of Pharmacy, Ain Shams University 11566-Abbassia Cairo Egypt; c Center for Drug Discovery Research and Development, Faculty of Pharmacy, Ain Shams University Cairo 11566 Egypt; d Department of Medical Biochemistry, Faculty of Medicine, Harran University Sanliurfa 63290 Türkiye; e Department of Biology, Faculty of Science, Khartoum University Khartoum Sudan sakinayagi@gmail.com; f Innovation Institute of Integrated Traditional Chinese and Western Medicine, Shandong First Medical University & Shandong Academy of Medical Sciences Jinan China limy@sioc.ac.cn

## Abstract

*Ecballium elaterium* is a perennial plant species that belongs to the *Cucurbitaceae* family. In this study, we examined the biological activities and analyzed the chemical profiles of ethyl acetate, acetone, aqueous acetone, and water extracts from different parts (aerial parts, fruit peels, and seeds) of *E. elaterium*. We performed chemical characterization using UPLC-ESI-MS/MS. To investigate the biological activities of the extracts, antioxidant capacity, enzyme inhibition, and cytotoxicity assays were conducted. Thirty-eight compounds were detected in the extracts, and cucurbitacins and their glycosides were the major components in the aerial parts and fruit peels. The aqueous acetone extract of the seeds exhibited the highest ABTS radical scavenging activity (155.82 mg TE per g), whereas the acetone extract of the aerial parts showed the greatest reducing power. The ethyl acetate extract of the aerial parts exhibited the highest inhibitory activity against AChE and BChE. Overall, the seed extracts showed glucosidase-inhibitory activity. The ethyl acetate extract of the aerial parts exhibited notable cytotoxic activity against the HCT-116 cell line (IC_50_ of 90.44 µg mL^−1^). Network pharmacology analysis linked the cucurbitacin-rich constituents to a converging set of cancer-related protein targets, and molecular docking predicted favorable binding affinities toward key hub proteins such as EGFR and STAT3, offering a preliminary computational rationale for the observed antitumor effects. As a result, the different *E. elaterium* extracts may serve as potential sources of bioactive compounds for further study and development in health-promoting applications.

## Abstract 2

*Ecballium elaterium* can be considered a bioactive raw material for developing future applications. Image created using Google Gemini.

## Graphical abstract

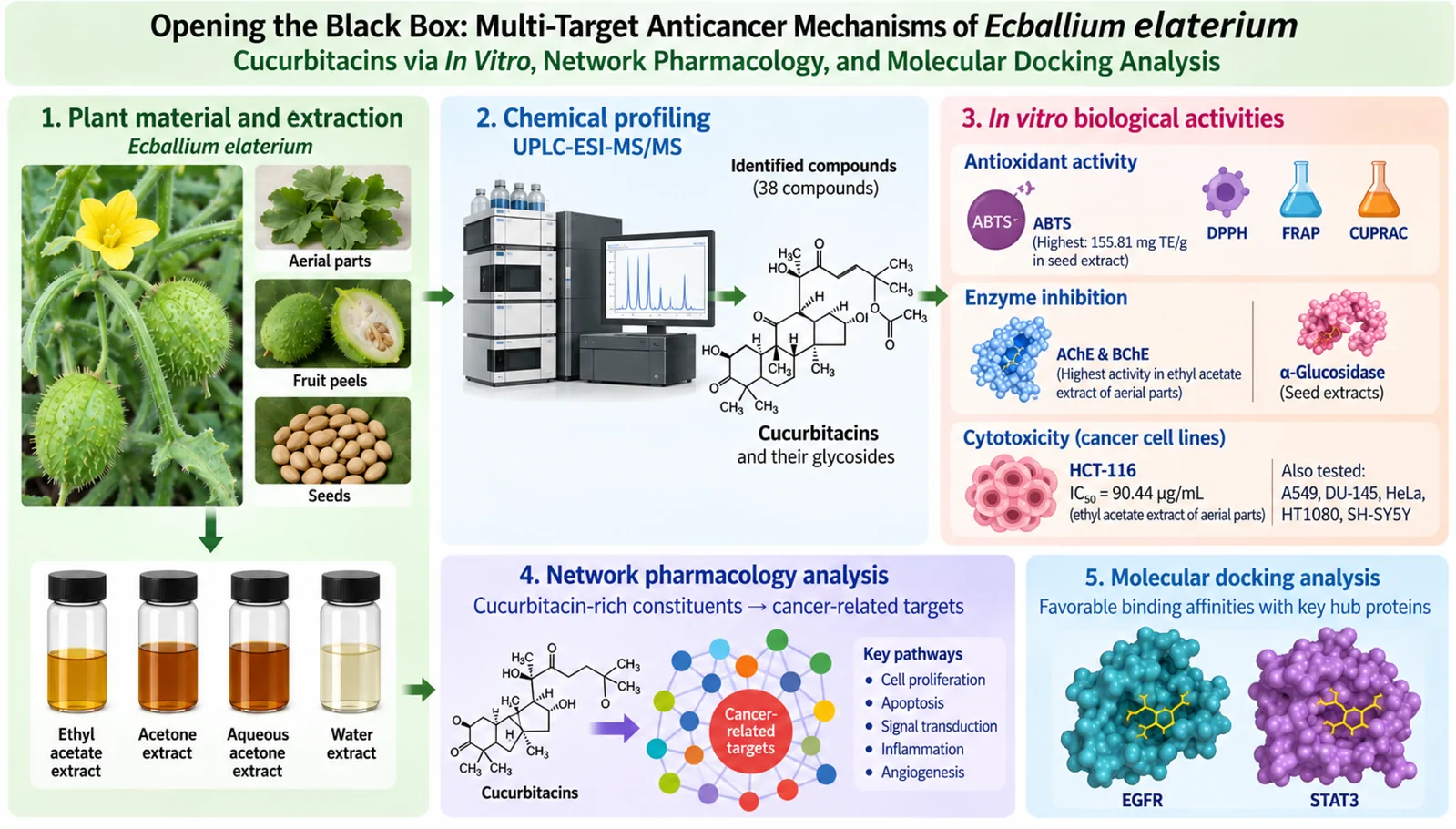

## 1 Introduction

The use of natural products for therapeutic purposes dates back to ancient civilizations. People across different cultures have used natural resources to prevent and treat diseases.^1,2^ Today, natural products remain widely used to treat many illnesses. They exhibit a variety of biological activities, including antioxidant, antidiabetic, and anticancer effects.^3^ Furthermore, natural products contribute to advances in pharmaceutical research; studies on natural products focus on developing new pharmaceutical agents.^4–6^ Plants contain a wide range of phytochemicals, known as secondary metabolites. These secondary plant metabolites encompass various chemical classes, including phenolic compounds, terpenes, sulfur-containing molecules, and nitrogen-based compounds.^7,8^ Several studies have demonstrated the antioxidant capacity of secondary metabolites and their enzyme-inhibiting effects associated with chronic diseases. Additionally, secondary metabolites enhance general health and protect against certain diseases.^9–11^ Given that medicinal plants are rich sources of secondary metabolites, they are widely used in drug development.^12,13^

*Ecballium elaterium*, known as the squirting cucumber, is a perennial plant species that belongs to the *Cucurbitaceae* family. It is native to the Mediterranean region and is also found in North Africa and West Asia.^14,15^ In traditional folk medicine, it has been used as a purgative and to treat various conditions, including chronic sinusitis, liver cirrhosis, rheumatism, infectious diseases, skin disorders, and pain.^16–19^*E. elaterium* contains a variety of constituents, including different cucurbitacins, sterols, flavonoids, terpenoids, fatty acids, and phenolic compounds. In addition these secondary metabolites, linoleic, punicic, and oleic acids are the main components in *E. elaterium* seeds.^20^ Cucurbitacins are the main components of *E. elaterium*.^14,21^ Because of cucurbitacins A, E, and I, the fruit and its juice are toxic. At high concentrations, they can cause symptoms affecting the respiratory, cardiovascular, and gastrointestinal systems.^15,17,18^ Despite *E. elaterium*'s toxicity, numerous studies have shown its antioxidant, antibacterial, and anticancer properties.^20,22^ Bourebaba *et al.* reported the antioxidant and anti-inflammatory potential of *E. elaterium* extracts.^14^ Touihri-Barakati *et al.* reported that the phenolic extract of *E. elaterium* inhibited adhesion and migration in HT1080 cells, indicating that it has the potential to suppress processes associated with metastasis.^23^ In another study, *E. elaterium* extract exhibited cytotoxic activity against SH-SY5Y and DU-145 cell lines.^17,24^ These findings emphasize the need for further investigation into the biological activities of *E. elaterium* extracts.

Although several biological activities of *E. elaterium* extracts have been studied, the work on their enzyme inhibitory activity, network pharmacology and molecular docking behavior is limited. Thus, the current study aimed to characterize the antioxidant properties of *E. elaterium* extracts by radical scavenging, reducing power, metal chelating capacity and phosphomolybdenum assay and their chemical composition by UPLC-MS/MS analysis. Enzyme inhibitory activities related to Alzheimer's disease, pigmentation disorders and diabetes were also studied. The cytotoxic activity of the extracts was evaluated on HeLa, A549, HCT-116 and HEK-293 cell lines using the MTT assay, Annexin V/PI assay and cell cycle analysis. Putative protein targets of the major constituents were then predicted by network pharmacology and their binding interactions with cancer-related proteins were evaluated by molecular docking, to provide mechanistic insights into the observed antitumor activity. These results pave the way for future studies on *E. elaterium* as a source of bioactive compounds with potential health applications.

## 2 Materials and methods

### 2.1 Plant collection

In October 2024, plant parts were collected from Selçuklu, Erenköy (coordinates: 37.908523, 32.461575), Konya, Turkey. Dr Evren Yıldıztugay carried out the taxonomic identification, and the corresponding voucher specimen is maintained in the herbarium of Selçuk University (Voucher number: ES-1001). The plant materials (seed, peel, and aerial parts) were separated, dried in the shade at room temperature, milled, and finally stored under dry, dark conditions.

### 2.2 Plant extract preparation

We extracted the plant parts (aerial parts, fruit peels, and seeds) using four solvents: ethyl acetate, acetone, aqueous acetone (70%), and water. Each sample (10 g) was macerated with 200 mL of ethyl acetate, acetone, or acetone/water for 24 hours at room temperature. We performed water extraction using the infusion method. The organic solvents were removed by rotary evaporation, and the aqueous extracts were lyophilized using a freeze-dryer.

### 2.3 Total phenolic and flavonoid contents

Total phenolic^25–27^ and total flavonoid^28^ contents in the extracts were determined using validated colorimetric methods, following established procedures. Calibration curves were prepared with gallic acid for phenolics and rutin for flavonoids, enabling the expression of the results as GAE and RE per gram, respectively.

### 2.4 UPLC/MS/MS analysis

We evaluated the phytochemical composition of *E. elaterium* extracts using the previously described method, which employed ultra-performance liquid chromatography (UPLC) coupled to an ESI-MS/MS detector.^34^ The compounds were tentatively identified based on their retention times, molecular ions, fragmentation patterns, and comparison with previously reported literature data. The confidence level of identification based on mass accuracy, fragmentation pattern, and literature precedent (Tiers A and B), where A confidence level means that the fragmentation pattern matches a specific literature spectrum for that exact compound and B confidence level means class-level only, exact identity undetermined.

### 2.5 Antioxidant assays

A set of *in vitro* assays were employed to evaluate the antioxidant activity, following the protocol described by ref. 29–32. The reducing power was determined using the FRAP and CUPRAC methods, while radical scavenging capacity was assessed by DPPH and ABTS assays. The outcomes of these four tests were converted into Trolox equivalents and reported as mg TE per gram of dried extract. In addition, the total antioxidant capacity was measured using the phosphomolybdenum (PBD) assay (mmol TE per g), and the metal chelating activity was expressed as mg EDTA equivalents per gram (mg EDTAE per g).

### 2.6. Enzyme inhibitory activity assays

Following established colorimetric methods,^29,33,34^ the extracts were evaluated for their ability to inhibit five key enzymes: acetylcholinesterase (AChE), butyrylcholinesterase (BChE), tyrosinase, α-amylase, and α-glucosidase. To ensure comparability among samples, inhibition results were expressed relative to appropriate reference compounds. Accordingly, cholinesterase inhibition was reported as mg GALAE per g, α-amylase and α-glucosidase inhibition as mmol ACAE per g, and tyrosinase inhibition as mg KAE per g.

#### 2.6.1 Cytotoxicity

##### 2.6.1.1 Cell culture

Three cancer lines and one normal line, all sourced from ATCC and kept in liquid nitrogen until use, were employed in this work: HeLa (cervical adenocarcinoma), A549 (lung adenocarcinoma), HCT-116 (colon adenocarcinoma), and HEK-293 (human embryonic kidney). Cells were grown in DMEM-F12/RPMI-1640 medium supplemented with 10% fetal bovine serum (FBS), 100 µg mL^−1^ streptomycin, and 100 IU mL^−1^ penicillin, and maintained at 37 °C in a humidified atmosphere with 5% CO_2_.

##### 2.6.1.2 Cell viability assay

The cytotoxicity of the *E. elaterium* extracts was determined by the MTT (3-(4,5-dimethylthiazol-2-yl)-2,5-diphenyltetrazolium bromide) assay. The cells were seeded into 96-well sterile plates at a density of 1 × 10^4^ cells per well and allowed to attach for 24 hours. The medium was then replaced with extract solutions covering a dose range of 2.5–200 µg mL^−1^, and the cells were exposed for a further 24 hours. The MTT reagent (10 µL, 0.5 mg mL^−1^) was added to each well; after 4 hours, the medium was discarded and 100 µL of DMSO was added to dissolve the formazan crystals. The absorbance was recorded at OD_570_–OD_690_ nm using a plate reader (Thermo Scientific Multiskan GO, Thermo, USA), and the obtained values were used to construct dose–response curves and calculate IC_50_.

##### 2.6.1.3 Apoptotic effect of the ethyl acetate extracts of the aerial parts and fruit peels on HCT-116 cells (morphological assessment)

Morphological hallmarks of apoptosis were examined in HCT-116 cells treated with the ethyl acetate extract of the aerial parts (90 µg mL^−1^) or the fruit peels (100 µg mL^−1^). After treatment, the cells were rinsed with PBS, fixed in 70% ethanol, washed again with distilled water, and stained with an acridine orange/ethidium bromide working solution (Cat. No. A6014-E1510, Sigma-Aldrich, Germany) before examination under a fluorescence microscope.

##### 2.6.1.4 Apoptotic effect of the ethyl acetate extracts of the aerial parts and fruit peels on HCT-116 cells (Annexin V/PI assay)

Apoptosis was further quantified using the FITC Annexin V Apoptosis Detection Kit I (BD Biosciences, New Jersey, USA), following the manufacturer's protocol. HCT-116 cells were seeded in 6-well plates (5 × 10^5^ cells per well), allowed to attach for 24 hours, and then exposed to the ethyl acetate extract of the aerial parts (90 µg mL^−1^) or the fruit peels (100 µg mL^−1^) for a further 24 hours. Cells were trypsinized, collected, and resuspended in 1× binding buffer, and then incubated for 15 minutes at room temperature. Fluorochrome-conjugated Annexin V (5 µL) and propidium iodide (5 µL) were added to each sample, followed by 100 µL of 1× binding buffer; the samples were centrifuged at 1200 rpm and incubated for 5 minutes before analysis using a BD flow cytometer (BD, New Jersey, USA).

##### 2.6.1.5 Cell cycle analysis of the ethyl acetate extract of the aerial parts on HCT-116 cancer cells

Cell cycle distribution was assessed using the BD Cycletest kit (New Jersey, USA), based on propidium iodide staining. HCT-116 cells were seeded at a density of 1–6 × 10^5^ cells per mL in 6-well sterile plates and, roughly one day later, treated with 90 µg mL^−1^ of the aerial-part extract for a further 24 hours. The cells were then detached with trypsin, pelleted by centrifugation (1500 rpm, 5 minutes), and resuspended; the pellet was washed twice with 1 mL PBS, treated with 1× binding buffer, and centrifuged again (1500 rpm, 5 minutes) to remove the supernatant. Solution A (trypsin, 250 µL) was added and the sample was incubated in the dark for 10 minutes with gentle mixing, followed by the addition of solution B (trypsin inhibitor, 200 µL) and incubation for another 10 minutes in the dark. Finally, 200 µL of solution C (propidium iodide) was added, and after further incubation in the dark for 10 minutes, the samples were analyzed by flow cytometry using a BD FACS instrument (BD, New Jersey, USA).

##### 2.6.1.6 Network pharmacology prediction

To gain mechanistic insights into the multitarget pharmacological profile of *E. elaterium*, we used a network-based *in silico* approach that integrated compound annotation with target prediction and functional enrichment. The chemical constituents previously identified *via* UPLC-ESI-MS/MS served as the ligand set for subsequent analyses. For each compound, we retrieved the corresponding three-dimensional structure from the PubChem database (https://pubchem.ncbi.nlm.nih.gov/) using its assigned Compound ID (CID), and predicted potential protein targets using the SwissTargetPrediction platform (https://swisstargetprediction.ch/). To ensure data reliability, we retained only targets with a probability score above zero and merged the resulting target lists from all compounds for further evaluation.

Concurrently, we systematically curated disease-associated genes from the GeneCards (https://www.genecards.org/) and OMIM (https://omim.org/) databases using the keywords “cervical cancer”, “lung adenocarcinoma”, and “colorectal cancer”, reflecting the primary bioactivities observed in our experimental assays. Following the removal of duplicate entries, the intersection between compound-predicted targets and disease-related genes was determined using the Venny 2.1 online tool (https://bioinfogp.cnb.csic.es/tools/venny/). We considered this overlapping gene set as putative therapeutic targets of *E. elaterium* and visualized it as a compound-target network in Cytoscape (version 3.8.0), scaling node sizes and color gradients by connectivity degree to highlight key regulators.

To further explore functional associations among these candidate targets, we constructed a protein–protein interaction (PPI) network using the STRING database (https://cn.string-db.org/), restricted to Homo sapiens and a high-confidence interaction score threshold >0.400. We discarded non-interacting nodes to improve the network clarity. The resultant PPI network was imported into Cytoscape for topological analysis, wherein the node degree was calculated using the “Analyze Network” module, and the nodes were graphically encoded with diameters proportional to their degree values (ranging from 60 to 150 pixels), facilitating the identification of hub proteins with central regulatory roles.

For functional annotation, the overlapping target genes were subjected to enrichment analysis using the Metascape platform (https://metascape.org/gp/index.html). This covered three Gene Ontology (GO) subcategories, biological processes (BP), molecular functions (MF), and cellular components (CC), as well as Kyoto Encyclopedia of Genes and Genomes (KEGG) pathway enrichment. Only terms with a *p*-value ≤0.05 were considered statistically significant. The top ten enriched terms within each category were ranked by enrichment score and summarized to delineate the principal signaling cascades and biological processes potentially modulated by the extracts.

##### 2.6.1.7 Molecular docking

To computationally explore the binding modes of the major bioactive constituents, molecular docking was performed against a set of relevant target proteins (EGFR, STAT3, BCL2, TNF, IL6, and HIF1A). The three-dimensional structures of the selected compounds were obtained from the PubChem database (https://pubchem.ncbi.nlm.nih.gov/) using their respective compound IDs. Before docking, each ligand was energy-minimized and conformationally refined with Chem3D v22.2.0 using the MM2 force field. Concurrently, the crystallographic coordinates of the target proteins were downloaded from the RCSB Protein Data Bank (https://www.rcsb.org/). All protein structures were preprocessed in PyMOL v3.1.6.1 and AutoDockTools (ADT) 1.5.7, during which water molecules and heteroatoms were removed, polar hydrogens were added, and Gasteiger charges were assigned. The protonation states were assigned at a physiological pH of 7.4. The docking grid boxes were centered at the active sites of each target, with the following coordinates and dimensions: EGFR (center: 83.452, 59.349, 59.183; size: 122.079, 101.085, 72.924), STAT3 (center: 0.227, 30.881, 33.273; size: 144.511, 75.659, 149.357), BCL2 (center: 5.94, 1.721, 9.845; size: 39.534, 37.222, 43.733), TNF (center: 20.246, 50.863, 41.168; size: 63.229, 64.34, 62.324), IL6 (center: 0.52, −19.83, 9.741; size: 46.205, 49.984, 41.001), and HIF1A (center: 21.101, 29.437, 28.175; size: 56.552, 65.17, 56.211). The docking calculations were then conducted using AutoDock Vina 1.2.0, with the exhaustiveness value set to 8 and the number of binding modes set to 9 within an energy range of 3 kcal mol^−1^.

##### 2.6.1.8 Statistical analysis

All experiments were conducted in triplicate, and the differences among the extracts were assessed for statistical significance. One-way ANOVA was conducted using GraphPad Prism (version 9.2), followed by Tukey's post hoc test for pairwise comparisons. A *p*-value less than 0.05 was considered statistically significant.

### 2.7. Declaration of generative AI and AI-assisted technologies in the writing process

During the preparation of this work, the author(s) used ChatGPT (OpenAI) and Grammarly to improve the clarity and language of the manuscript. In addition, the authors used Google Gemini for designing the graphical abstract. Following the use of this tool, the authors carefully reviewed and revised all content as necessary and accept full responsibility for the final published article.

## 3 Results

### 3.1. Total phenolic and total flavonoid contents

Several studies have reported a significant correlation between the total phenolic and flavonoid contents and antioxidant capacity.^35^Table 1 shows the total phenolic (TPC) and total flavonoid (TFC) contents in extracts obtained from different parts of *E. elaterium*. The acetone extracts of the aerial parts and fruit peels had the highest phenolic content, measuring 32.26 mg GAE per g and 24.60 mg GAE per g, respectively. In contrast, the aqueous acetone extracts of the aerial parts and fruit peels contained the lowest phenolic content (9.61 GAE per g and 5.34 GAE per g, respectively). Among the seed extracts, the ethyl acetate extract had the highest phenolic content (16.08 mg GAE per g). In terms of flavonoid content, the highest level was observed in the ethyl acetate extract of fruit peels with 16.69 mg RE per g. In aerial parts and seeds, the acetone/water extracts contained more flavonoids than the ethyl acetate and water extracts. Mejri *et al.* reported the total phenolic content in the ethyl acetate and water extracts of *E. elaterium* leaves as 3.58 mg GAE per g and 5.01 mg GAE per g, respectively.^36^ In another study by Felhi *et al.*, the total phenolic contents of the acetone extracts of *E. elaterium* seeds and fruit peels were reported as 34 mg GAE per g and 44 mg GAE per g, respectively.^22^ In contrast, the present study observed lower values for the acetone extracts of the fruit peel and seeds. These differences may be due to variations in locality, climate, or solvent-polarity efficiency.^37,38^

**Table 1:** Extraction yields (%), total phenolic content (TPC) and total flavonoid content (TFC) in various solvent extracts of *E. elaterium*^a^

Parts	Solvents	Extraction yields (%)	TPC (mg GAE per g)	TFC (mg RE per g)
Aerial parts	EA	2.88	29.07 ± 0.29^b^	1.84 ± 0.24^ef^
	Acetone	2.16	32.26 ± 0.09^a^	2.10 ± 0.24^de^
	Acetone/water	19.79	9.61 ± 0.07^i^	6.17 ± 0.09^de^
	Water	19.34	10.22 ± 0.15^h^	2.41 ± 0.07^c^
Fruit peels	EA	3.25	20.41 ± 0.35^d^	16.69 ± 0.44^a^
	Acetone	2.84	24.60 ± 0.12^c^	11.24 ± 0.31^b^
	Acetone/water	20.53	5.34 ± 0.09^k^	1.54 ± 0.16^f^
	Water	12.66	6.79 ± 0.05^j^	0.84 ± 0.07^g^
Seeds	EA	19.78	16.08 ± 0.22^e^	0.35 ± 0.06^gh^
	Acetone	19.10	11.04 ± 0.34^g^	0.14 ± 0.01^g^
	Acetone/water	13.44	12.47 ± 0.19^f^	0.69 ± 0.05^gh^
	Water	1.53	12.09 ± 0.22^f^	0.40 ± 0.04^gh^

a The values are reported as mean ± SD of three parallel measurements. TPC: total phenolic content; TFC: total flavonoid content; GAE: gallic acid equivalent; RE: rutin equivalent. Different letters indicate significant differences between the tested extracts (*p* < 0.05).

### 3.2. Metabolic profiling of different parts *of E. elaterium*

The dried matter of different parts of *E. elaterium*, including aerial parts, fruit peels, and seeds, was extracted using ethyl acetate, acetone, acetone/water (70%), and water solvents. The dried extracts were then subjected to UPLC-ESI-MS/MS analysis for a comprehensive metabolic profiling of the plant. The tentatively identified metabolic compounds are summarized in Table 2.

**Table 2:** Metabolic profiling of different parts of *E. elaterium* (AP: aerial part; FP: fruit peels; S: seed; EA: ethyl acetate; AC: acetone; and Wat: water)^c^

No.	*R*_t_	Name	[M + H]^+^	[M − H]^−^	ms ms^−1^	Confidence level	AP-EA	AP-Ac	AP-Ac/Wat	AP-Wat	FP-EA	FP-Ac	FP-Ac/Wat	FP-Wat	S-EA	S-Ac	S-Ac/Wat	S-Wat	Reference
1	0.62	Caffeic acid derivative		377	**179**	A	−	−	−	+	−	−	−	−	−	−	−	−	43
2	0.8	Trehalose^a^		377	356, 350, 338, 267, 131, **89**	A	−	−	+	+	−	−	+	−	−	−	−	+	17
3	0.865	Citric acid		191	111, 87	A	−	−	−	−	−	−	−	+	−	−	−	−	17
4	1.16	Phenyl alanine	166		119, 131	A	−	−	+	−	−	−	−	−	−	−	−	−	41
5	5.686	Shikimic acid		173	137, 93	A	−	−	−	−	−	−	−	−	+	+	+	−	40
6	5.707	Aconitic acid		173	111, 85	A	−	−	−	−	−	−	−	−	+	+	+	−	79
7	6.113	Methyl suberic acid		187	125	A	−	−	−	−	−	−	−	−	+	+	−	−	42
8	6.42	Rutin	611	609	301	A	−	−	+	−	−	−	+	+	−	−	−	−	43
9	6.768	Azelaic acid		187	**181**, **143**, **125**, **97**	A	−	−	−	−	−	−	−	−	+	+	+	−	40
10	7.383	Bryoamaride^b^		723	677, 685, 619	A	−	−	−	+	−	−	+	+	−	−	−	+	39
11	7.385	Cucurbitacin S	499		**499**, **383**, **149**, **137**, **123**, **113**	A	−	−	+	+	+	+	+	+	−	−	−	−	17
12	7.419	Cucurbitacin I 2-O-β-d-glucopyranoside^b^		721	**513**, 632, 589, 580, 460, 389, 379	A	−	+	+	+	+		+	+	−	−	−	−	39
13	7.428	Khekadaengoside D		721	**675**, **598**	A	−	+	+	+	+	+	−	+	−	−	−	−	17
14	7.464	Arvenin III		723	**659**, 599, 550, 475, 436, 433	A	−	−	+	+	+	+	+	+	−	−	−	−	17
15	7.643	Kuguacin G	501		**483**, **465**, **401**, **369**, **341**, **335**, **283**	A	−	−	−	−	+	+	+	+	−	−	−	−	39
16	7.88	Unknown ursane-type triterpenoid glycoside^b^		707	**88**, **100**, **309**, **549**, **571**	B	−	+	+	+	−	−	−	−	−	−	−	−	17
17	8.464	Arvenin I		765	**701**, **659**, **630**, **555**, **113**	A	−	−	−	−	+	+	+	−	−	−	−	−	17
18	8.674	Khekadaengoside K		561	**399**, **443**, **504**, **521**	A	−	−	−	+	+	−	−	+	−	−	−	−	39
19	8.937	Trihydroxy octadecenoic acid		329	**311**, **171**	A	−	−	+	+	−	−	−	−	+	+	+	+	42
20	8.972	Cucurbitacin E 2-O-β-d-glucopyranoside^b^		763	**717**, **552**, **527**, **387**, **289**	A	−	−	−	−	+	+	+	−	−	−	−	−	39
21	9.051	Kuguacin F	483		**483**, **448**, **441**, **373**, **323**, **321**	A	−	−	−	−	−	−	+	−	−	−	−	−	39
22	9.033	Cucurbitacin F^b^		563	**517**, **385**, **165**	A	−	−	−	+	+	+	+	+	−	−	−	+	39
23	9.11	Arvenin II^b^		767	**481**, **647**, **707**	A	−	−	−	+	+	+	+	+	−	−	−	−	17
24	9.24	Oxo-dihydroxy-octadecenoic acid		327	**211**, **171**	A	−	+	+	+	+	−	+	−	+	+	−	+	80
25	9.504	22-Deoxocucurbitoside B^b^		835	**789**, **730**, **652**, **578**, **505**, **194**	A	−	−	−	−	+	+	+	−	−	−	−	−	39
26	9.834	Seco-cucurbitacin B derivative		589	**550**, **515**, **493**, **331**, **135**	A	−	−	−	−	+	+	−	−	−	−	−	−	17
27	10.37	Cucurbitacin I		559	565, 550, 540, **498**	A	−	+	+	+	+	+	+	+	−	−	−	−	17
28	11.255	Cucurbitacin B^b^		603	497, 481, 463, 439, 408, 316, 274	A	−	−	−	−	+	+	+	+	−	−	−	−	17
29	12.186	Cucurbitacin E^b^		601	495, 484, 410, 347	A	−	+	−	−	+	+	−	−	−	−	−	−	17
30	15.473	Hydroxyoctadecadienoic acid		295	277, 195	A	−	+	−	−	+	+	+	−	+		+	+	42
31	16.11	Hydroxy oleic acid		297	279	A	−	−	−	−	−	−	−	−	+	+	+		41
32	17.547	Octadecatrienoic acid	279		165, 149, 135, 123, 107, 95, 83, 69	A	−	−	−	−	−	−	−	−		+	+		40
33	18.83	Octadecadienamide	280		263, 246, 209, 179, 163, 147, 133	A	−	+	+	−	+	+	+	+	−	−	+	+	42
34	20.2	Stearidonic acid		277	259	A	+	−	−	+	+	+	−	−	−	−	−	−	42
35	20.718	Esculin		339	162	A	−	−	−	−	−	−	+	−	+	−	−	+	81
36	20.388	Hexadecanamide	256		242, **130**, **116**	A	+	+	+	+	+	+	+	+	+	+	+	+	42
37	20.903	Oleamide	282		**265**	A	+	+	+	+	+	+	+	+	+	+	+	+	42
38	23.6	Stearic acid	284		200, 186, 167, 144, 125	A	+	+	+	+	+	+	+	+	+	+	+	+	43

a [M + Cl]^−^.

b [M + HCOOH-H].

c A: A confidence level: fragmentation pattern matches a specific literature spectrum for that exact compound. B: B confidence level: class-level only, exact identity undetermined.

The tentative identification of the compounds was based on their retention time for each compound, molecular ions, and fragments, and the comparison of the obtained data with those published previously. To ensure extensive chemical characterization, both positive and negative modes were applied.^17,39^

A total of 38 compounds were tentatively annotated, and they belonged to different classes, including organic acids, amino acids, phenolics and caffeic acid derivatives, flavonoids, cucurbitacins and their glycosides, triterpenoids, and fatty acids/amides. The negative ion mode provided a broader view of chemical diversity and was more informative for compound annotation. The major identified class in aerial parts and fruit peels was cucurbitacins, and their glycosides; the fragmentation pattern was in agreement with previous reports. For instance, cucurbitacin I ([M + FA-H]^−^ at *m*/*z* 559), E ([M + FA-H]^−^ at *m*/*z* 601), and F ([M + FA-H]^−^ at *m*/*z* 563) were detected in both aerial parts and peels. Cucurbitacin B ([M + FA-H]^−^ at *m*/*z* 603) and seco-cucurbitacin B derivative were detected only in fruit peels.^17^ Moreover, a few glycosides were annotated, such as cucurbitacin I 2-O-β-d-glucopyranoside ([M + FA-H]^−^ at *m*/*z* 721).^39^

Organic acids, such as shikimic and citric acids ([M − H]^−^ at *m*/*z* 173 and 191, respectively), were annotated.^17,40^ One disaccharide, trehalose ([M − H]^−^ at *m*/*z* 377), was observed in acetone/water and water extracts of all parts of the plant.^17^

Meanwhile, seed extracts showed fatty acids as the predominant compounds, such as trihydroxy octadecenoic acid ([M − H]^−^ at *m*/*z* 329), hydroxyoctadecadienoic acid ([M − H]^−^ at *m*/*z* 295) and hydroxy oleic acid ([M − H]^−^ at *m*/*z* 297).^41,42^ Only one flavonoid was present: rutin ([M − H]^−^ at *m*/*z* 609) in acetone/water and water extracts of both aerial parts and fruit peels.^43^

In the positive mode, fatty acids/amides were predominant. For example, hexadecanamide, oleamide, and stearic acid ([M + H]^+^ at *m*/*z* 256, [M + H]^+^ at *m*/*z* 282, and [M + H]^+^ at *m*/*z* 284, respectively) were present in all parts.^42,43^ Moreover, cucurbitacin S ([M + H]^+^ at *m*/*z* 499) was detected in aerial parts and fruit peels.^17^ Noteworthy, class cucurbitacin and their glycosides were exclusively previously tentatively identified in *E. elaterium*.^17^

### 3.3. Antioxidant effects

The antioxidant capacity of *E. elaterium* extracts was evaluated using various *in vitro* assays: DPPH, ABTS, CUPRAC, FRAP, phosphomolybdenum total antioxidant capacity (PPM), and metal chelating activity (MCA). Table 3 shows the antioxidant effects of the different extracts obtained from different parts of *E. elaterium.* In the DPPH assay, the aqueous acetone extract of the aerial parts showed the highest activity (38.70 mg TE per g). In contrast, the lowest DPPH activity was recorded for the ethyl acetate extract of the fruit peels (6.88 mg TE per g). The ethyl acetate and acetone extracts of the seeds showed no DPPH scavenging activity. In the ABTS assay, the aqueous acetone extract of the seeds showed the highest activity (155.81 mg TE per g). Conversely, the acetone extract of the seeds showed the lowest activity (3.41 mg TE per g). The CUPRAC and FRAP assays showed that the acetone extracts of the aerial parts had the highest reducing power (101.28 mg TE per g and 56.16 mg TE per g, respectively). However, the aqueous extract of the fruit peels and the acetone extract of the seeds showed the lowest reducing power (17.74 mg TE per g and 21.93 mg TE per g, respectively). In the metal chelating assay, the highest chelating ability was found in the aqueous acetone extract of the seeds (22.32 mg EDTA per g), while the lowest activity was recorded for the ethyl acetate extract of the fruit peels (1.68 mg EDTA per g). Phosphomolybdenum results showed that the acetone extract of the aerial parts exhibited the best activity (2.18 mmol TE per g), whereas the aqueous extract of the aerial parts had the lowest activity (0.02 mmol TE per g). The presence of various compounds in the extracts, such as rutin, cucurbitacin derivatives (B, E, F, I, and S), and citric acid, may contribute to the antioxidant activity. Several studies have reported the antioxidant potential of these compounds.^44–46^*In vitro* studies have shown that cucurbitacin derivatives exert significant antioxidant activity through multiple mechanisms. Studies on cucurbitacin B and E glucosides (CGC) have demonstrated a dose-dependent ability to scavenge several reactive oxygen species (ROS), including hydroxyl radicals (˙OH), superoxide anions (O_2_˙^−^) and singlet oxygen (^1^O_2_). The respective IC values measured by ESR spectroscopy were 0.38, 8 and 11 mM. The combination also reduced preformed ABTS˙^+^ to its native form and inhibited MDA formation during linoleic acid oxidation in a dose-dependent manner.^47^

**Table 3:** Antioxidant properties of the *E. elaterium* extracts^a^

Parts	Solvents	DPPH (mg TE per g)	ABTS (mg TE per g)	CUPRAC (mg TE per g)	FRAP (mg TE per g)	MCA (mg EDTAE per g)	PPM (mmol TE per g)
Aerial parts	EA	23.09 ± 0.97^cd^	84.66 ± 1.58^e^	74.25 ± 2.26^b^	50.29 ± 0.48^b^	20.32 ± 0.56^b^	2.10 ± 0.09^a^
	Acetone	26.97 ± 0.28^b^	103.03 ± 2.13^d^	101.28 ± 9.29^a^	56.16 ± 5.34^a^	17.37 ± 0.30^c^	2.18 ± 0.08^a^
	Acetone/water	38.70 ± 2.37^a^	78.39 ± 2.47^f^	37.48 ± 0.18^de^	41.16 ± 1.41^c^	7.43 ± 0.93^f^	0.48 ± 0.13^ef^
	Water	25.55 ± 1.38^bc^	120.81 ± 1.70^c^	33.31 ± 1.72^e^	36.19 ± 0.88^cde^	16.85 ± 0.20^c^	0.02 ± 0.01^g^
Fruit peels	EA	6.88 ± 0.53^f^	67.50 ± 2.46^g^	54.64 ± 1.93^c^	39.69 ± 1.39^cd^	1.68 ± 0.23^h^	1.51 ± 0.03^bc^
	Acetone	9.51 ± 1.80^ef^	79.55 ± 1.09^ef^	47.90 ± 2.36^c^	37.42 ± 0.67^cde^	9.79 ± 0.67^e^	1.54 ± 0.14^bc^
	Acetone/water	12.15 ± 0.59^e^	41.39 ± 1.55^h^	32.05 ± 0.78^e^	32.80 ± 0.71^e^	4.78 ± 0.29^g^	0.33 ± 0.01^f^
	Water	10.88 ± 0.89^e^	63.39 ± 1.40^g^	17.74 ± 0.10^f^	23.75 ± 0.14^f^	11.83 ± 0.12^d^	0.54 ± 0.04^ef^
Seeds	EA	na	4.04 ± 0.76^i^	54.90 ± 3.72^c^	24.11 ± 0.23^f^	20.85 ± 0.27^ab^	1.34 ± 0.09^c^
	Acetone	na	3.41 ± 0.96^i^	45.19 ± 2.88^cd^	21.93 ± 0.68^f^	16.01 ± 1.62^c^	0.92 ± 0.05^d^
	Acetone/water	22.31 ± 0.34^d^	155.81 ± 2.62^a^	54.79 ± 3.01^c^	55.49 ± 2.47^ab^	22.32 ± 0.05^a^	1.66 ± 0.11^b^
	Water	20.71 ± 0.46^d^	127.64 ± 1.97^b^	28.42 ± 0.36^e^	34.83 ± 0.94^de^	11.33 ± 0.17^de^	0.59 ± 0.03^e^

a The values are reported as mean ± SD of three parallel measurements. PPM: phosphomolybdenum; MCA: metal chelating activity; TE: trolox equivalent: EDTAE: EDTA equivalent; and na: not active. Different letters indicate significant differences between the tested extracts (*p* < 0.05).

The total antioxidant capacity, reducing power and anti-ROS/RNS activity of cucurbitacin E glucoside and its newly synthesised esterified derivatives—cinnamyl and caffeoyl conjugates—were evaluated. The esterified derivatives showed more pronounced anti-inflammatory, total antioxidant, reducing power, anti-ROS, and anti-RNS activities than the parent compound. The caffeoyl derivative (which bears catechol groups) showed the greatest potency, which is consistent with the well-established role of *ortho*-dihydroxyphenyl groups in radical scavenging.^48,49^

Previous studies have also shown that cucurbitacin B and cucurbitacin I have antioxidant properties and can inhibit lipid peroxides.^50^ Overall, these *in vitro* findings demonstrate that cucurbitacin derivatives act as multi-target antioxidants, combining direct free radical scavenging with indirect upregulation of the body's natural antioxidant defences. The activity of these derivatives is modulated by key structural features, including the presence of free hydroxyl groups, an α,β-unsaturated ketone system, glycosylation and the nature of any conjugated aromatic acid moiety.

In a study reported by Mejri *et al.*, the antioxidant capacity of *E. elaterium* was assessed using the DPPH assay; the ethyl acetate extracts of the flower and fruit exhibited IC_50_ values of 223.33 µg mL^−1^ and 313.36 µg mL^−1^, respectively.^36^ Felhi *et al.* reported that the acetone extracts of the fruit peels and seeds showed ABTS radical scavenging activity with IC_50_ values of 1.1 mg mL^−1^ and 1.8 mg mL^−1^, respectively.^22^ Pearson's correlation analysis showed that the total phenolic content positively correlated with CUPRAC, FRAP, and phosphomolybdenum assays (Fig. 1). These results may be due not only to phenolic compounds but also to non-phenolic compounds such as vitamin C and tocopherol, which may be responsible for the observed antioxidant activity.^51,52^

**Fig. 1:**
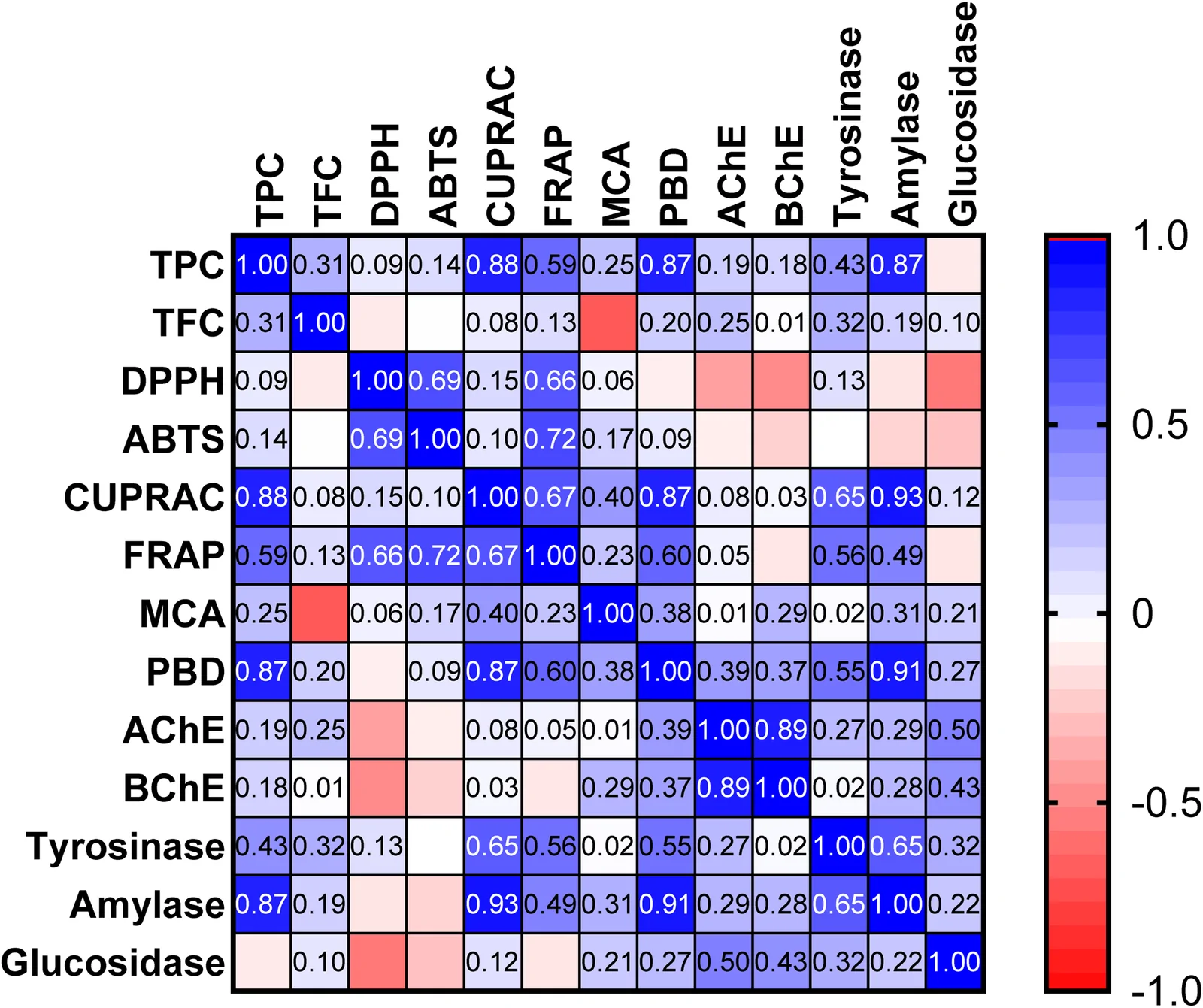
Pearson's correlation analysis (MCA: metal chelating assay and PBD: phosphomolybdenum).

### 3.4. Enzyme inhibitory effects

The different parts of *E. elaterium* extracts were tested against AChE, BChE, tyrosinase, amylase and glucosidase. The results are presented in Table 4. For AChE and BChE inhibition, the ethyl acetate extracts of the aerial parts showed the highest inhibition, with values of 1.89 mg GALAE per g and 4.63 mg per GALAE per g, respectively. Moreover, the lowest inhibition was observed in the aqueous acetone extracts of the aerial parts (AChE: 0.66 mg GALAE per g and BChE: 0.97 mg GALAE per g). Regarding tyrosinase inhibition, the highest inhibition was observed in the acetone extracts of the aerial parts and fruit peels, as well as the aqueous acetone extract of the seeds, with values of 65.95, 61.50, and 64.82 mg KAE per g, respectively. However, the water extract displayed weaker tyrosinase inhibition in the tested plant parts. The strongest amylase inhibition was obtained in the acetone extracts of the aerial parts (1.02 mmol ACAE per g), followed by the ethyl acetate (0.96 mmol ACAE per g) and the ethyl acetate extracts of the seeds (0.66 mmol ACAE per g). In the glucosidase inhibition, the aqueous acetone extract of the seeds exhibited the highest inhibition (7.54 mmol ACAE per g), followed by the acetone extracts of the seeds (6.98 mmol ACAE per g), the ethyl acetate extract of the fruit peels (6.58 mmol ACAE per g), and the ethyl acetate extract of the seeds (5.66 mmol ACAE per g). The presence of cucurbitacin derivatives can be attributed to the observed enzyme inhibitory effects of *E. elaterium*. For example, Sharma and Sharma investigated the AChE inhibitory properties of cucurbitacin B and E, which were isolated from *Trichosanthes dioica*, and revealed that cucurbitacin B and E exhibited concentration-dependent AChE inhibition, reaching 72% and 80% inhibition, respectively, at 100 µg mL^−1^.^53^ In another study by Oh *et al.*, the tyrosinase inhibitory activity of cucurbitacin derivatives obtained from *Trichosanthes kirilowii* was demonstrated, and these derivatives exhibited lower IC_50_ values of 0.18 and 6.7 µM, compared with hydroquinone (11.5 µM).^54^ Chang *et al.* also reported that cucurbitacin B, which obtained *Cucumis sativus*, showed anti-melanogenic activity by decreasing the activity in B16F1 melanoma cells.^55^ In another study, Lu *et al.* investigated the α-glucosidase inhibitory activity of compounds isolated from the roots of *Siraitia grosvenorii*; among them, cucurbitacin B showed a modest activity, with 50.59% inhibition at 1 mg mL^−1^.^56^ The literature contains only a small number of studies examining the enzyme inhibitory properties of *E. elaterium* extracts. In an earlier work, Orhan *et al.*^57^ evaluated the AChE, BChE, and tyrosinase inhibitory activities of ethanol extracts prepared from the fruit and aerial parts of *E. elaterium*. Neither extract showed AChE inhibitory activity. In the BChE assay, the fruit extract demonstrated 15.9% inhibition, while the aerial part extract showed 8.16% inhibition. In addition, the fruit extract produced a 6.18% tyrosinase inhibition effect, whereas the aerial part extract exhibited no tyrosinase inhibitory activity.

**Table 4:** Enzyme inhibitory effects of the *E. elaterium* extracts^a^

Parts	Solvents	AChE (mg GALAE per g)	BChE (mg GALAE per g)	Tyrosinase (mg KAE per g)	Amylase (mmol ACAE per g)	Glucosidase (mmol ACAE per g)
Aerial parts	EA	1.89 ± 0.19^a^	4.63 ± 0.07^a^	54.77 ± 3.75^e^	0.96 ± 0.02^a^	na
	Acetone	na	na	65.95 ± 2.50^a^	1.02 ± 0.07^a^	na
	Acetone/water	0.66 ± 0.01^d^	0.97 ± 0.07^e^	61.70 ± 0.29^abc^	0.29 ± 0.02^e^	na
	Water	na	0.98 ± 0.07^e^	47.10 ± 0.33^f^	0.08 ± 0.01^f^	na
Fruit peels	EA	1.79 ± 0.15^a^	2.55 ± 0.42^cd^	59.54 ± 1.71^cd^	0.65 ± 0.03^b^	6.58 ± 0.09^c^
	Acetone	1.86 ± 0.10^a^	3.37 ± 0.57^bc^	61.50 ± 0.81^bc^	0.60 ± 0.04^bc^	na
	Acetone/water	0.98 ± 0.04^c^	1.66 ± 0.15^de^	59.41 ± 0.14^cd^	0.30 ± 0.01^e^	na
	Water	na	1.39 ± 0.13^e^	37.20 ± 0.61^g^	0.07 ± 0.01^f^	na
Seeds	EA	1.40 ± 0.05^b^	3.28 ± 0.58^bc^	55.56 ± 0.91d^e^	0.66 ± 0.03^b^	5.66 ± 0.08^d^
	Acetone	1.66 ± 0.05^a^	3.55 ± 0.56^b^	57.08 ± 0.74d^e^	0.55 ± 0.03^cd^	6.98 ± 0.30^b^
	Acetone/water	1.84 ± 0.11^a^	3.28 ± 0.34^bc^	64.82 ± 0.21^ab^	0.47 ± 0.01^d^	7.54 ± 0.23^a^
	Water	1.76 ± 0.02^a^	2.82 ± 0.01^bc^	42.98 ± 0.03^f^	0.08 ± 0.01^f^	na

a The values are reported as mean ± SD of three parallel measurements. GALAE: galantamine equivalent; KAE: kojic acid equivalent; ACAE: acarbose equivalent; and na: not active. Different letters indicate significant differences between the tested extracts (*p* < 0.05).

### 3.5. Cytotoxic effect

The cytotoxic effects of *E. elaterium* extracts were tested on three different cancer cell lines (HeLa, A549, and HCT-116) and a normal cell line (HEK-293) to compare their effects on normal and cancer cell lines (Table 5). All ethyl acetate extracts demonstrated the highest cytotoxic effects on the cell lines. The ethyl acetate extracts of the aerial parts exhibited the lowest IC_50_ value (90.44 µg mL^−1^) in HCT-116 cells, while the ethyl acetate extracts of the fruit peels exhibited high cytotoxicity (94.1 µg mL^−1^). However, the ethyl acetate extracts of the aerial and fruit peels displayed cytotoxicity in normal HEK-293 cells (118.24 µg mL^−1^ and 101.17 µg mL^−1^, respectively). These results revealed that the extracts had weak selectivity between cancer and normal cell lines. Overall, the seed extracts showed the highest IC_50_ values. The seed water extracts showed weaker cytotoxic activity (1076.9–1212.56 µg mL^−1^). When comparing the cytotoxic activity of the plant parts, the fruit peel extracts showed the lowest IC_50_ values, followed by the aerial part and seed extracts. In addition, the aqueous acetone and water extracts of the fruit peels showed lower IC_50_ values than the same extracts of the aerial parts and seeds. In a study by Stefi *et al.*, the cytotoxic effects of the extracts were evaluated on the MCF-7, DU-145 and SH-SY5Y cell lines. At a concentration of 1000 µg mL^−1^, the extracts exhibited approximately 54%, 42%, and 67% cytotoxicity, respectively.^17^ To quantify tumour selectivity, selectivity indices (SI = IC_50_[HEK-293]/IC_50_[cancer line]) were calculated for the active extracts (Table 6). The SI values ranged from 0.65 to 1.31 and remained at or below the threshold of 2 that is conventionally taken to indicate selective cytotoxicity,^58^ the highest value being that of the aerial-part ethyl acetate extract against HCT-116 cells (SI = 1.31). These results show that, in their crude form, the extracts exert comparable cytotoxicity toward malignant and non-tumorigenic cells, and thus, lack pronounced tumour selectivity. This behaviour is consistent with the recognised broad-spectrum cytotoxicity and narrow therapeutic window of cucurbitacin-type triterpenoids,^59^ and indicates that the cucurbitacin-rich constituents identified here are more appropriately regarded as leads for subsequent purification and structural optimisation than as selective anticancer agents in the extract form.

**Table 5:** Cytotoxic effects of the *E. elaterium* extracts on cancer and normal cell lines (IC_50_ [µg mL^−1^])

Parts	Solvent	HELA	A549	HCT-116	HEK-293
Aerial parts	EA	121.58	158.21	90.44	118.24
	Acetone	147.29	201.51	149.56	131.31
	Acetone/water	779.55	870.55	851.89	728.33
	Water	871.12	820.28	766.71	770.38
Fruit peels	EA	101.18	99.17	94.1	101.17
	Acetone	133.33	158.12	115.72	114.18
	Acetone/water	351.77	312.63	390.51	313.21
	Water	309.92	384.24	385.84	317.16
Seeds	EA	590.23	601.89	580.03	543.43
	Acetone	1100.12	1096.39	923.78	912.39
	Acetone/water	985.47	1000.48	838.55	812.81
	Water	1212.56	1154.67	1103.1	1076.9

**Table 6:** Selectivity indices of the *E. elaterium* extracts (SI = IC_50_(HEK-293)/IC_50_(cancer line)). Seed extracts and all aqueous-acetone/water extracts (IC_50_ > 300 µg mL^−1^) were not considered active and were excluded from the analysis

Plant part	Solvent	HeLa	A549	HCT-116
Aerial parts	Ethyl acetate	0.97	0.75	1.31
	Acetone	0.89	0.65	0.88
Fruit peels	Ethyl acetate	1.00	1.02	1.08
	Acetone	0.86	0.72	0.99

Previous studies have demonstrated the cytotoxic effects of cucurbitacins B, E, and I against HCT-116 cells and of arvenin I against MCF-7 cells.^17,60^ Therefore, the presence of cucurbitacins (B, E, F, I, and S) and arvenins (I and III) in the ethyl acetate extracts of the fruit peels may contribute to cytotoxic effects. For instance, Chen *et al.* (2024) examined the cytotoxic activities of several cucurbitacins isolated from *Aquilaria sinensis* and found that some compounds—particularly cucurbitacin Q1—showed strong effects against cancer cell lines. They also reported that a 3α-hydroxyl group enhances bioactivity and that the α,β-unsaturated ketone (C-22 to C-24) in the cucurbitacin side chain markedly increases cytotoxic potency.^61^ In another study, Alafnan *et al.* (2022) reported that cucurbitacin B showed anticancer activity against prostate cancer PC-3 cell lines by regulating the JAK1/STAT1 signaling pathway and triggering apoptosis.^62,63^ Silva *et al.* also reported that cucurbitacin B showed significant cytotoxic potential against lung cancer by modulating programmed cell death.^64^ In another study, Jayaprakasam *et al.* (2003) reported that cucurbitacin I suppressed cell proliferation in colon cancer (HCT-116), breast cancer (MCF-7), lung cancer (NCI-H460), and astrocytoma (SF-268) cell lines.^65^

Additionally, to investigate the apoptotic effects of the ethyl acetate extract of the aerial parts in HCT-116 cells, AO/EB staining and Annexin-V/PI assays were conducted. The AO/EB fluorescent staining method is widely used to assess the cell viability and detect forms of cell death, such as apoptosis and necrosis.^66^ The Annexin V/PI staining method is widely used based on the binding interaction between Annexin V and phosphatidylserine, which is located in the extracellular layer of apoptotic and necrotic cells.^67^ In the Annexin V/PI assay, the ethyl acetate extract of the aerial parts induced early apoptosis (*X*%), late apoptosis (7.7%) and necrosis (39.7%) in HCT-116 cells at 90 µg mL^−1^, whereas the fruit-peel extract induced early apoptosis (*Y*%), late apoptosis (5.7%) and necrosis (61.4%) at 100 µg mL^−1^ (Fig. 2). These distributions indicate that the extracts trigger a mixed mode of cell death in which apoptotic and necrotic mechanisms operate concurrently, with the necrotic component predominating—most markedly for the fruit-peel extract—at the near-IC_50_ concentrations tested. Such a profile is consistent with the progression of a fraction of apoptotic cells to secondary necrosis during prolonged *in vitro* exposure in the absence of phagocytic clearance,^68^ and with the capacity of cucurbitacins to engage regulated necrotic programmes (necroptosis and pyroptosis) alongside apoptosis.^69–72^ The concurrent apoptosis-associated morphology observed by AO/EB staining, together with the ordered G0/G1 arrest (41.5% → 77.1%) detected by cell-cycle analysis, indicates that a regulated, non-passive component contributes to the observed cytotoxicity (Fig. 3). Jafargholizadeh *et al.* found that various cucurbitacin compounds isolated from *E. elaterium* triggered sub-G1 accumulation and induced cell death in AGS cells.^73^

**Fig. 2:**
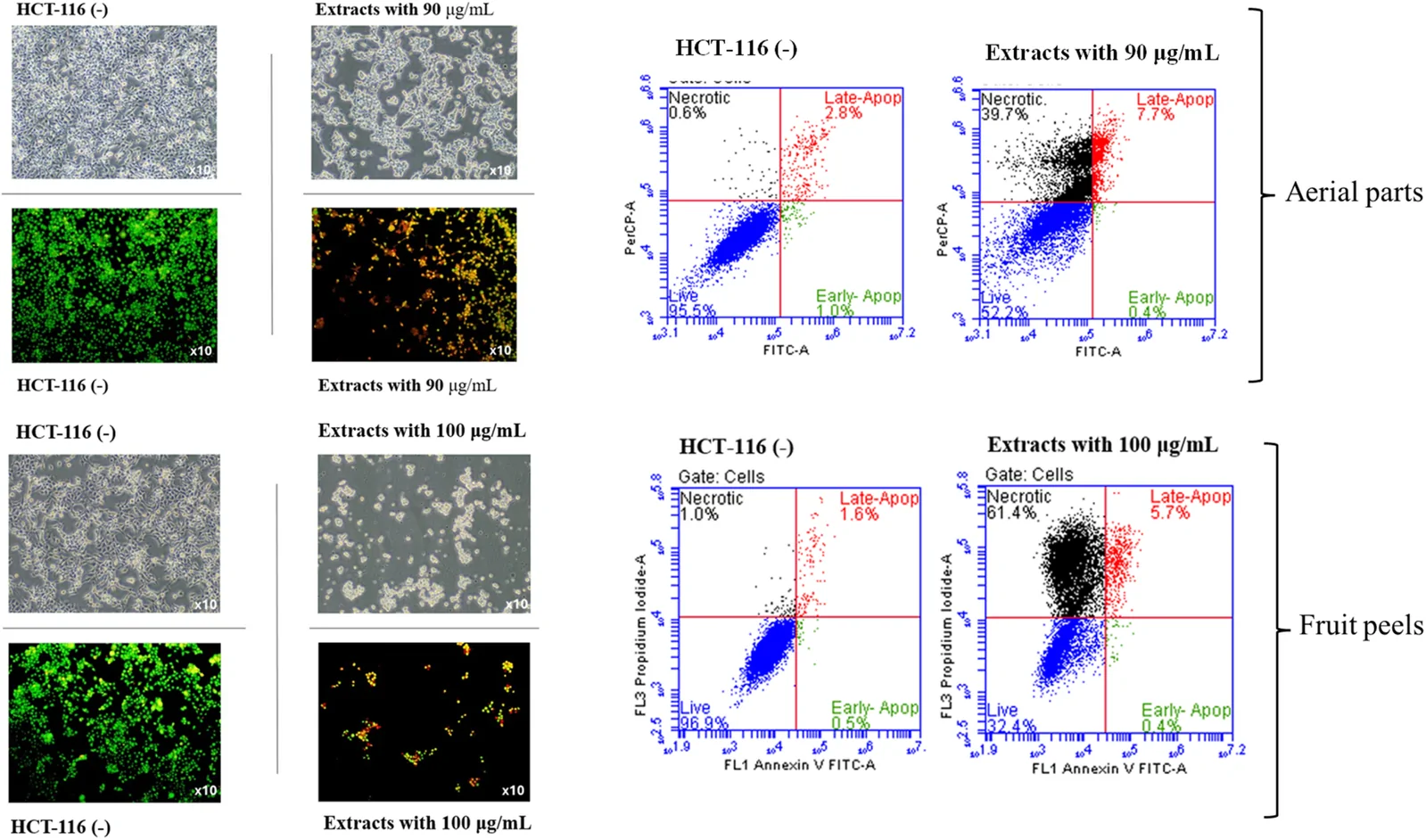
AO/EB staining after the ethyl acetate extracts of the aerial parts (90 µg mL^−1^) and fruit peels (100 µg mL^−1^) were applied to HCT-116 cells. Annexin-V/PI staining results after the ethyl acetate extract of the aerial parts (90 µg mL^−1^) and the fruit peels (100 µg mL^−1^) were applied to HCT-116 cells. Blue: live-[(FITC^−^)/(PI^−^)]; green: early apoptotic [(FITC^+^)/(PI^−^)]; red: late apoptotic [(FITC^+^)/(PI^+^)]; and black: necrotic [(FITC^+^)/(PI^+^)] cells.

**Fig. 3:**
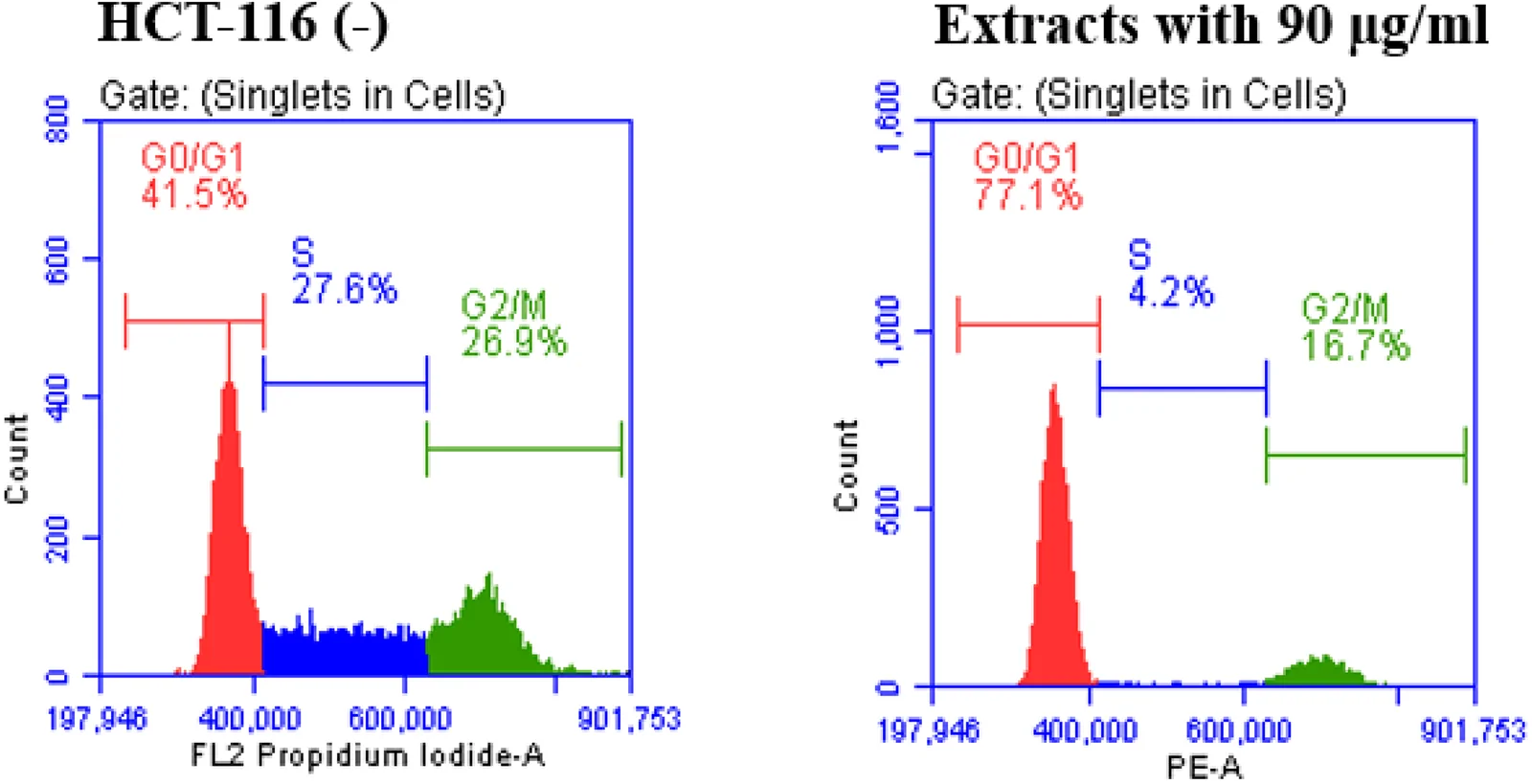
Cell cycle staining results after applying the ethyl acetate extract of the aerial parts (90 µg mL^−1^) to HCT-116 cells. Red: G0/G1 phase; blue: S phase; and green: G2/M phase.

### 3.6. Network pharmacology analysis

To explore the potential molecular mechanisms underlying the antitumor effects of *E. elaterium* extracts, a network pharmacology approach was employed. A total of 14 representative compounds were selected for target prediction based on two criteria. The first criterion was their high relative abundances in the bioactive extracts, particularly the ethyl acetate and acetone fractions of aerial parts and fruit peels, which demonstrated potent cytotoxicity *in vitro*. The second criterion was their representation of the major chemical classes identified, with emphasis on cucurbitacins and their glycoside derivatives, which are well-documented as hallmark bioactive constituents of *E. elaterium*. The selected compounds comprised 14 cucurbitacin-type triterpenoids, namely, cucurbitacins B, E, F, I, and S; their glycosylated congeners comprising cucurbitacin I 2-O-β-d-glucopyranoside and cucurbitacin E 2-O-β-d-glucopyranoside; and structurally related derivatives including arvenins I to III, bryoamaride, kuguacins F and G, khekadaengoside K, and 22-deoxocucurbitoside B.^74^ Target prediction using the SwissTargetPrediction platform yielded 245 non-redundant putative targets for these compounds. Concurrently, disease-associated genes for cervical cancer, lung adenocarcinoma, and colorectal cancer, corresponding to the HeLa, A549, and HCT-116 cell lines evaluated in the cytotoxicity assays, were retrieved from the GeneCards and OMIM databases, comprising 1,508, 1,055, and 1732 targets, respectively. The intersection between compound-predicted targets and each cancer-specific gene set revealed 61 common targets for lung adenocarcinoma, 81 for colorectal cancer, and 75 for cervical cancer. Notably, a core set of 53 targets was commonly shared across all three cancer types (Fig. 4A and B).

**Fig. 4:**
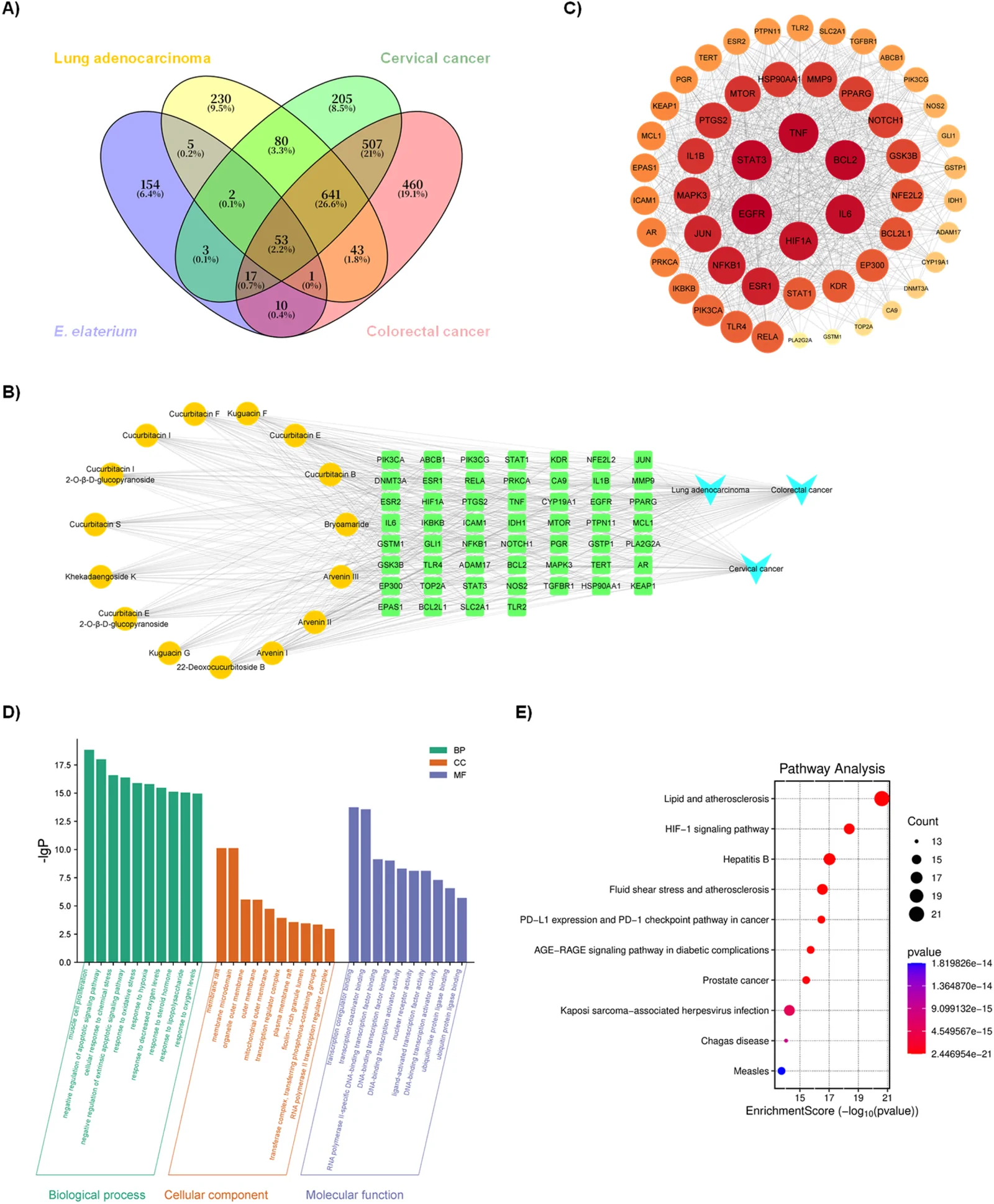
Network pharmacology and functional enrichment analysis of the *E. elaterium* extracts in prostatic cancer, cervical cancer and lung cancer. (A) Venn diagram shows 53 common targets of compounds from the *E. elaterium* extracts in lung adenocarcinoma, cervical cancer and colorectal cancer. (B) Component-disease-target network diagram. (C) PPI network of 53 co-targeted genes/proteins. (D) GO enrichment analysis results for the anti-cancer targets. (E) Top 10 KEGG pathways enriched.

To further explore interconnectivity among these 53 common targets, we constructed a protein–protein interaction network using the STRING database with a high-confidence interaction threshold above 0.400. The resulting network comprised 53 nodes interconnected by 832 edges, indicating a densely connected topology. Topological analysis in Cytoscape showed an average node degree of 31.4, substantially higher than expected by chance, suggesting that these targets form a highly integrated signaling network. The network showed characteristic hub nodes with high degree centrality, including EGFR (degree 50), STAT3 (degree 49), BCL2 (degree 49), TNF (degree 49), IL6 (degree 48), and NFKB1 (degree 45). These hub proteins are well-established as central nodes in cancer signaling networks, governing cell survival, proliferation, apoptosis, and inflammatory responses. The betweenness centrality analysis further highlighted EGFR, TNF, and STAT3 as key bridge nodes with values of 87.86, 94.72, and 59.45, respectively, underscoring their critical roles in mediating crosstalk among different signaling pathways. Conversely, several peripheral nodes including GSTM1, PLA2G2A, and CA9 exhibited lower degrees of connectivity, suggesting more specialized or context-dependent functions. The identification of multiple highly interconnected hub nodes is consistent with the view that the 15 cucurbitacin-dominant compounds may exert their antitumor effects through synergistic modulation of a tightly integrated protein interaction network rather than through isolated single–target interactions. However, this synergistic hypothesis requires experimental validation using appropriate functional assays (Fig. 4C).

To further characterize the functional roles of the 53 common targets, gene ontology enrichment analysis was performed, covering biological process, cellular component, and molecular function categories. The most significantly enriched biological processes were predominantly associated with cellular stress responses and the regulation of cell death pathways. Specifically, muscle cell proliferation, negative regulation of apoptotic signaling pathway, cellular response to chemical stress, and response to oxidative stress emerged as the top-ranked terms, with enrichment scores of 18.84, 17.99, 16.58, and 15.88, respectively. Other highly enriched processes included response to hypoxia, response to steroid hormone, and response to lipopolysaccharide, all of which are intimately linked to the tumor microenvironment and cancer progression. The prominence of oxidative stress and apoptosis-related terms is particularly noteworthy, as it shows consistency with experimental observations that ethyl acetate extracts of aerial parts and fruit peels induced late apoptosis and G0/G1 phase arrest in HCT-116 cells. Taken together, these *in silico* and experimental results raise the possibility that cucurbitacin-rich constituents may exert their cytotoxic effects by activating stress-response pathways and tipping the balance of apoptotic signaling toward cell death. Nevertheless, direct evidence for the involvement of these specific pathways in the observed cell death requires further experimental investigation. Molecular function analysis showed that the 53 common targets were significantly enriched in transcription-related activities. The top-ranked molecular functions included transcription coregulator binding, transcription coactivator binding, and RNA polymerase II-specific DNA-binding transcription factor binding, with enrichment scores of 13.75, 13.56, and 9.13, respectively. Other enriched functions such as nuclear receptor activity, ubiquitin-like protein ligase binding, and DNA-binding transcription activator activity further underscored the regulatory nature of these targets. This transcriptional regulation signature is consistent with the known mechanism of cucurbitacins, which according to previous reports modulate the activity of key transcription factors including STAT3, NF-κB, and p53, thereby influencing the expression of genes involved in cell cycle progression, apoptosis, and inflammation. However, whether the specific compounds identified in our extracts indeed modulate these transcription factors in the tested cell lines remains to be determined experimentally. The cellular component analysis indicated that the targets were predominantly localized to membrane-associated structures and transcription regulatory complexes. Membrane raft and membrane microdomain were the most significantly enriched terms with enrichment scores of 10.13, followed by organelle outer membrane and mitochondrial outer membrane. The enrichment of mitochondrial outer membrane components is particularly relevant, given the central role of mitochondria in the intrinsic apoptotic pathway. This observation corroborates the experimental findings that the extracts induced apoptosis in HCT-116 cells, as mitochondrial membrane permeabilization is a critical early event in apoptosis execution. Additionally, the enrichment of transcription regulator complex and RNA polymerase II transcription regulator complex further supports the transcriptional regulatory role of these targets (Fig. 4D).

KEGG pathway enrichment analysis was subsequently performed to identify the signaling cascades most significantly associated with the 53 common targets (Fig. 4E). The results revealed that these targets were predominantly enriched in pathways related to lipid metabolism and atherosclerosis, cancer signaling, infectious diseases, and immune regulation. The most significantly enriched pathway was Lipid and atherosclerosis with an enrichment score of 20.61, followed by the HIF-1 signaling pathway with a score of 18.38, Hepatitis B with a score of 17.02, and fluid shear stress and atherosclerosis with a score of 16.54. The prominence of atherosclerosis-related pathways may initially appear surprising in the context of cancer research; however, these pathways share core inflammatory and oxidative stress components that are critically involved in both cardiovascular diseases and tumorigenesis. The enrichment of HIF-1 signaling pathway is particularly noteworthy, given its central role in cellular adaptation to hypoxic conditions, a hallmark of the tumor microenvironment.^75^ HIF-1 activation promotes angiogenesis, metabolic reprogramming, and survival under low oxygen tension, all of which contribute to cancer progression and therapeutic resistance. The convergence of multiple cucurbitacin-rich compound targets on this pathway offers a computational basis for hypothesizing that these extracts may exert their antitumor effects in part by interfering with hypoxia-adaptive responses.

The enrichment of PD-L1 expression and PD-1 checkpoint pathway in cancer is particularly intriguing as it raises the hypothesis that the cucurbitacin constituents may modulate immune checkpoint signaling, potentially enhancing antitumor immune responses. However, this immunomodulatory hypothesis is purely speculative at this stage and requires dedicated experimental testing, such as assessment of PD-L1 expression levels in treated cancer cells or co-culture studies with immune cells. The enrichment of prostate cancer (score 15.43) and pancreatic cancer (score 12.51) further underscores the broad-spectrum anticancer potential of these compounds. Notably, the PI3K-Akt signaling pathway, one of the most frequently dysregulated pathways in human cancers, was also significantly enriched with a score of 12.36. This pathway regulates cell survival, proliferation, and metabolism, and its inhibition has been implicated in the anticancer effects of various cucurbitacins. The enrichment of EGFR tyrosine kinase inhibitor resistance is also noteworthy, as it prompts the hypothesis that cucurbitacin derivatives may circumvent or overcome resistance to conventional targeted therapies. Again, this possibility remains to be experimentally verified using appropriate drug-resistant cell line models.

Immune and inflammatory pathways were also prominently represented in the KEGG enrichment results. TNF signaling pathway, IL-17 signaling pathway, Th17 cell differentiation, and Toll-like receptor signaling pathway were all significantly enriched with scores ranging from 12.24 to 13.22. These pathways are intimately linked to the tumor microenvironment and chronic inflammation, which are well-established drivers of carcinogenesis. The convergence of multiple targets on these immune-related pathways suggests that the antitumor effects of *E. elaterium* extracts may involve both direct cytotoxicity and immunomodulatory mechanisms. Furthermore, pathways related to infectious diseases, including Hepatitis B, Toxoplasmosis, Measles, and Coronavirus disease-COVID-19, were also enriched, reflecting the shared host cellular machinery that viruses and cancers exploit for survival and replication. The identification of apoptosis as a significantly enriched pathway with a score of 8.45 is consistent with the experimental flow cytometry results, wherein the ethyl acetate extracts induced late apoptosis in HCT-116 cells. Similarly, the enrichment of necroptosis with a score of 4.55 aligns with the observation of necrosis in the Annexin-V/PI assay, particularly for the fruit peel extracts. This consistency between the KEGG pathway predictions and the experimental cytotoxicity data supports the potential relevance of the identified targets and generates testable hypotheses for future mechanistic studies. However, it should be noted that pathway enrichment based on computational target prediction does not constitute experimental proof that these pathways are indeed modulated in the treated cells.

The integration of PPI network topology with GO and KEGG enrichment results offers a coherent *in silico* model, wherein the 15 selected compounds are predicted to target a densely interconnected network of proteins that converge on the regulation of apoptotic signaling, oxidative stress responses, transcriptional control, and immune modulation. The identification of 53 common targets shared across all three cancer types is consistent with the possibility that the antitumor activity of *E. elaterium* may operate, at least in part, through a conserved set of molecular hubs implicated in oncogenic processes. This observation aligns with the known ability of cucurbitacins to modulate fundamental cellular functions including cell cycle progression, apoptosis, and signal transduction that are dysregulated across diverse malignancies. The convergence of these targets on apoptosis and necroptosis pathways provides a plausible computational rationale for the observed cytotoxicity of the ethyl acetate and acetone extracts against all three cancer cell lines, suggesting that the cucurbitacin-rich composition may exert multi-target effects on core oncogenic pathways. The predominance of cucurbitacin derivatives among the 15 selected compounds, which were primarily derived from the most biologically active extraction fractions, is consistent with a close association between the chemical profile and the pharmacological outcomes. The multi-target nature of these compounds, as reflected by the 245 predicted targets and their substantial overlap with cancer-associated genes, raises the hypothesis that the observed antiproliferative effects may arise from synergistic interactions among multiple constituents acting on interconnected signaling networks. This aligns with the broader understanding that cucurbitacins may function through the simultaneous modulation of multiple pathways involved in cell survival, apoptosis evasion, and therapeutic resistance. The weak selectivity observed between cancer and normal HEK-293 cells may be tentatively explained by the fact that these core pathways are also essential for normal cellular homeostasis, highlighting the need for future structural optimization or targeted delivery strategies to improve the therapeutic index of cucurbitacin-based agents. Importantly, all of these computational observations should be considered hypothesis-generating and require rigorous experimental validation, such as western blotting, qPCR, or target-specific inhibition studies, to confirm the predicted pathway modulations in the tested cell lines.^76^

### 3.7. Molecular docking

To computationally explore the binding interactions between the major bioactive constituents of *E. elaterium* and their potential therapeutic targets, we performed molecular docking simulations. Based on the network pharmacology analysis, we selected six core target proteins as receptors for docking studies: EGFR, STAT3, BCL2, TNF, IL6, and HIF1A. We selected these proteins because of their high degree and betweenness centrality within the PPI network, indicating their pivotal roles as hub nodes in cancer-related signaling pathways. A total of 12 representative compounds were selected as ligands, comprising cucurbitacins B, E, F, I, S, their glycosylated derivatives including cucurbitacin E 2-O-β-d-glucopyranoside, and structurally related compounds including arvenins I to III, 22-deoxocucurbitoside B, and kuguacins F and G. These compounds were selected based on their abundance in the most bioactive extracts and their established presence in the UPLC-ESI-MS/MS metabolic profiling.

The docking simulations predicted favorable binding energies for all tested compounds across the six target proteins, with values ranging from −6.6 to −10.4 kcal mol^−1^. Generally, binding energies below −7.0 kcal mol^−1^ are considered indicative of strong ligand–protein interactions, suggesting potential favorable binding of these cucurbitacin derivatives toward the selected oncogenic targets. Notably, 22-deoxocucurbitoside B showed the lowest predicted binding energy toward EGFR with a value of −10.2 kcal mol^−1^, while cucurbitacin E 2-O-β-d-glucopyranoside exhibited the strongest predicted affinity for STAT3 (−10.4 kcal mol^−1^), representing the most favorable predicted interaction observed across all ligand–target pairs. The glycosylated derivatives, particularly cucurbitacin E 2-O-β-d-glucopyranoside and 22-deoxocucurbitoside B, consistently exhibited superior binding energies compared to their aglycone counterparts, suggesting that glycosylation may enhance molecular recognition and binding complementarity with these protein targets. This observation aligns with previous reports, indicating that the sugar moiety can contribute to hydrogen bonding networks and overall binding stability (Fig. 5). However, these computational predictions should be interpreted with caution, as they represent *in silico* estimates that require experimental corroboration.

**Fig. 5:**
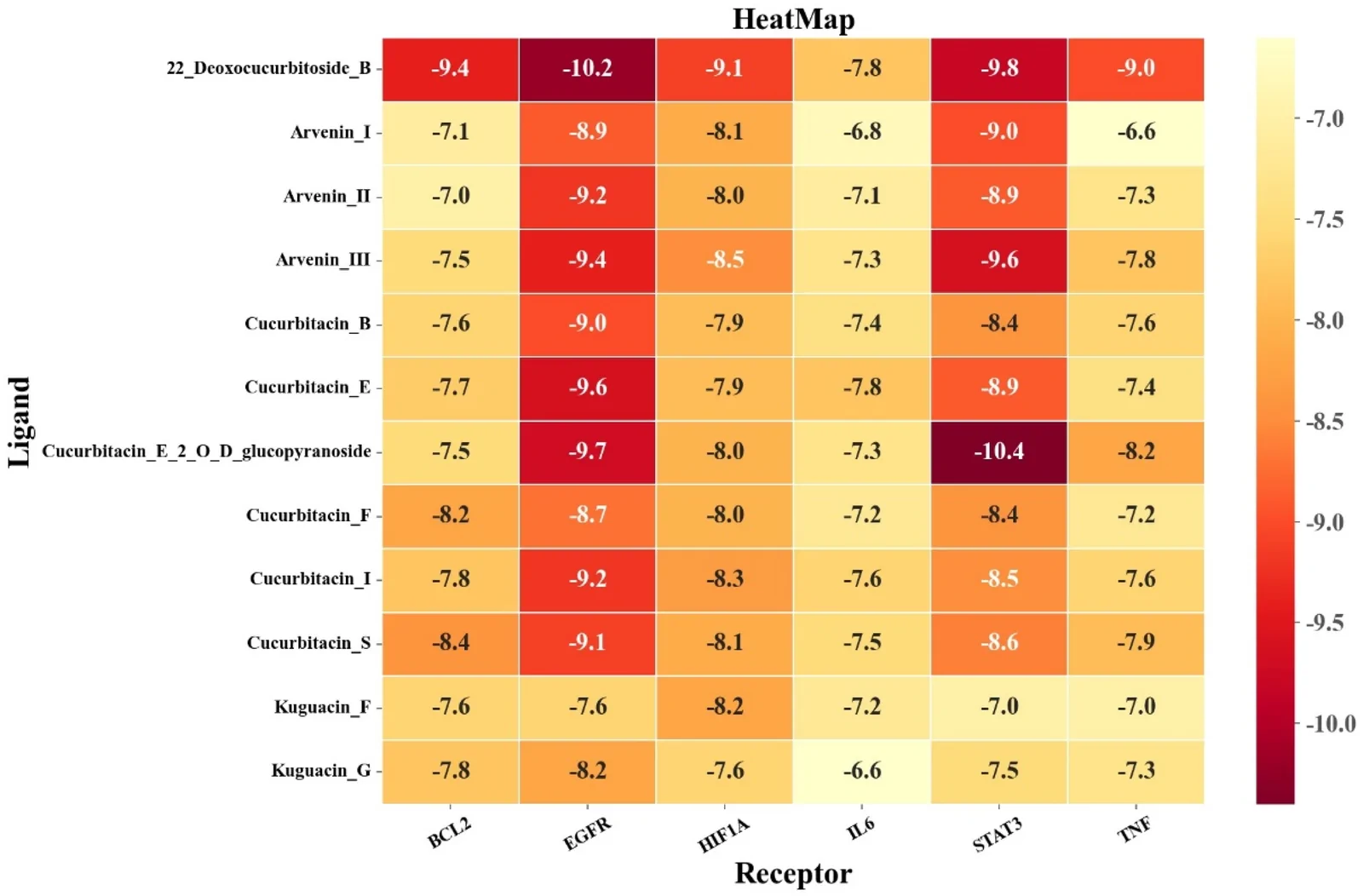
Docking score heatmap (Δ*G*, kcal mol^−1^) of 12 compounds from *E. elaterium* against six cancer-related target proteins.

Among the target proteins, STAT3 and EGFR consistently exhibited the lowest binding energies across most ligands, with average values of −8.8 and −9.0 kcal mol^−1^, respectively, suggesting that these two proteins are particularly susceptible to modulation by cucurbitacin-type compounds. This finding is consistent with their established roles as major oncogenic drivers and the known ability of cucurbitacins to interfere with STAT3 phosphorylation and EGFR signaling. However, direct inhibition of these targets by the specific compounds identified in our extracts requires further experimental verification. In contrast, IL6 and TNF, primarily inflammatory cytokines, showed weaker binding affinities (average values of −7.3 and −7.5 kcal mol^−1^, respectively), indicating that while these targets remain relevant, the compounds may exert anti-inflammatory effects through alternative mechanisms or require higher concentrations for effective modulation.

A clear structure–activity relationship can be discerned from the docking results. Cucurbitacin E 2-O-β-d-glucopyranoside and 22-deoxocucurbitoside B, both bearing glycosidic moieties, consistently outperformed their non-glycosylated counterparts across all tested targets, highlighting the importance of sugar conjugation for enhanced target engagement. Among the aglycones, cucurbitacin S and cucurbitacin F demonstrated relatively stronger binding, with average values of −8.3 and −7.9 kcal mol^−1^, respectively, likely due to their unique hydroxylation patterns and the presence of α,β-unsaturated ketone systems that facilitate hydrogen bonding and hydrophobic interactions within the binding pockets. The arvenin series exhibited moderate predicted binding affinities ranging from −6.6 to −9.6 kcal mol^−1^, with arvenin III showing consistently stronger interactions than arvenins I and II, suggesting that subtle structural variations may influence target selectivity and potency.

Furthermore, the docking predictions are consistent with the experimental cytotoxicity data, in which the ethyl acetate extracts of aerial parts and fruit peels, enriched in these cucurbitacin derivatives, showed the lowest IC_50_ values against HCT-116, HeLa, and A549 cells. The predicted affinity toward BCL2 and EGFR, both of which are critical regulators of apoptosis and cell proliferation, offers a plausible computational rationale for the observed induction of apoptosis and G0/G1 phase arrest in the flow cytometry analyses. However, these *in silico* observations should be interpreted with caution and require experimental verification. In particular, the predicted interaction with BCL2, an anti-apoptotic protein, is consistent with the notion that the extracts may neutralize BCL2 function, potentially tipping the apoptotic balance toward cell death. Nevertheless, direct binding affinity and functional inhibition of BCL2 by these compounds require experimental validation. Overall, these *in silico* findings provide preliminary structural insights that are suggestive of potential interactions between cucurbitacin derivatives and core oncogenic targets, thereby supporting the network pharmacology predictions and offering a computational framework for understanding the observed biological activities of *E. elaterium* extracts (Fig. 6).^77,78^ However, given the inherent limitations of docking-based approaches, these predictions should be regarded as hypothesis-generating and warrant further experimental investigation.

**Fig. 6:**
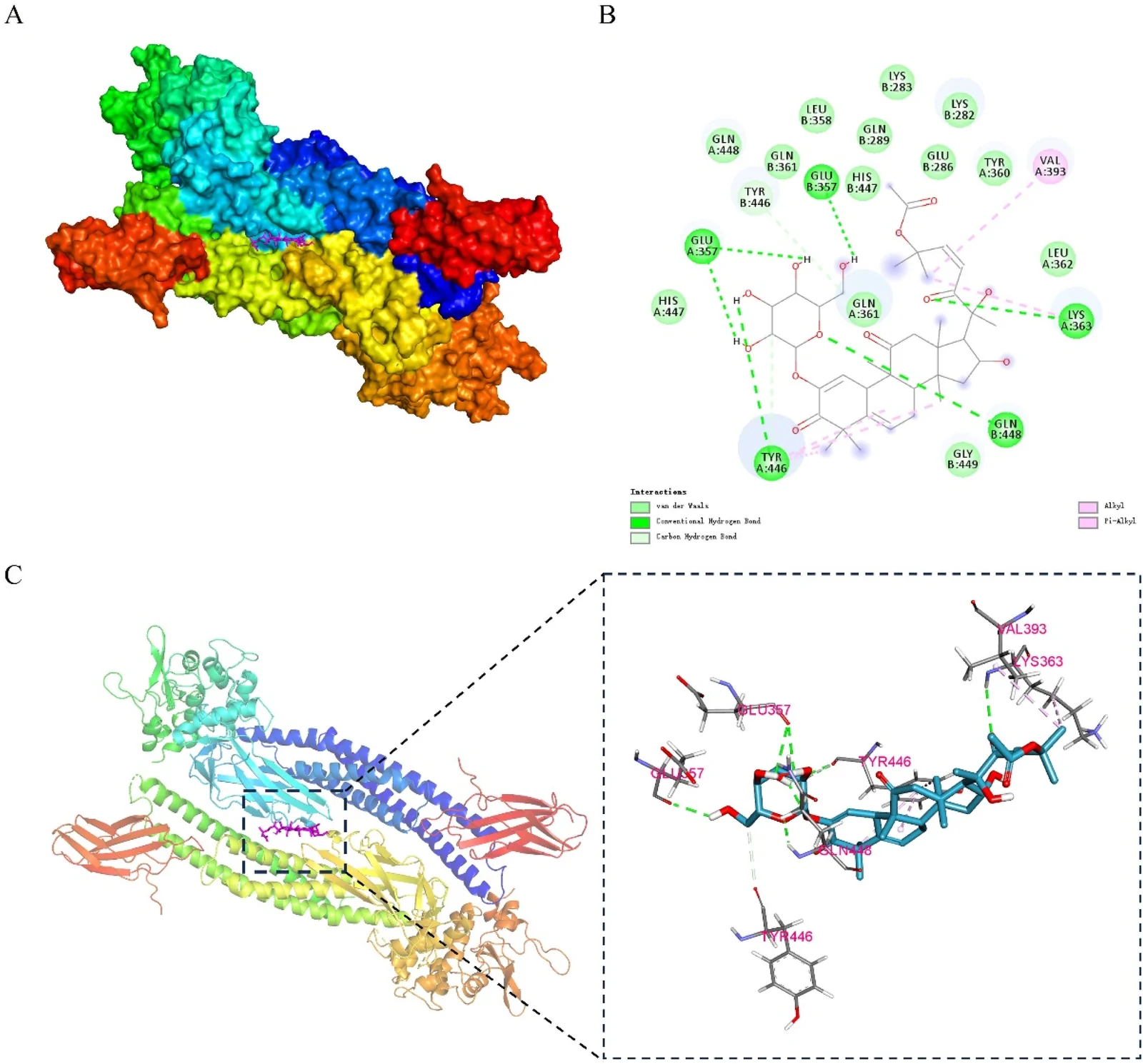
Schematic of molecular docking between STAT3 and cucurbitacin E 2-O-β-d-glucopyranoside. (A) Binding mode of cucurbitacin E 2-O-β-d-glucopyranoside in the STAT3 active site. (B) 2D Representation of the binding pockets of STAT3 with cucurbitacin E 2-O-β-d-glucopyranoside. (C) 3D Schematic of the combined site.

## 4 Conclusion

Overall, the present study disclosed the qualitative phytochemical profiles of *E. elaterium* extracts by UPLC-ESI-MS/MS and comprehensive biological activity, including antioxidant, enzyme-inhibitory, and cytotoxic activity assays. The UPLC-ESI-MS/MS revealed the main chemical groups to be cucurbitacins and cucurbitacin glycosides. TPC correlated with the CUPRAC, FRAP, and PBD tests. In general, the extracts from aerial parts showed a higher reducing power than that of the seed extracts. However, the seed extracts showed more AChE, BChE and glucosidase inhibition than the aerial part extracts. Although the ethyl acetate extracts of the aerial parts and fruit peels displayed the most pronounced cytotoxicity (IC_50_ ≈ 90–94 µg mL^−1^ against HCT-116), their selectivity indices approximated unity, indicating limited discrimination between cancer and normal cells; the principal value of these extracts therefore lies in delineating cucurbitacin-based chemical scaffolds and their putative molecular targets for further development rather than in direct therapeutic application. Consistent with these findings, network pharmacology analysis identified a core group of cancer-related targets shared by the major cucurbitacin components. Molecular docking studies predicted promising binding affinities for hub proteins such as EGFR and STAT3, offering preliminary *in silico* insights that may help explain the observed cytotoxic and apoptotic effects. These computational results, however, should be considered hypothesis-generating and require further experimental validation. These results position *E. elaterium* extracts as a promising source of bioactive compounds for pharmaceutical and cosmetic use. Their cytotoxicity toward HEK-293 cells and limited selectivity, however, mean that isolating the active compounds and defining dose-dependent effects across cancer *versus* normal cell lines will be the essential next step.

## Conflicts of interest

The authors declare no conflicts of interest.

## Abbreviations

ACAE: Acarbose equivalents
ABTS: 2,2′-Azino-bis(3-ethylbenzothiazoline-6-sulfonic acid)
AChE: Acetylcholinesterase
ATCC: American type culture collection
A549: Human lung adenocarcinoma cell line
BCHE: Butyrylcholinesterase
BP: Biological process
CC: Cellular component
CUPRAC: Cupric ion reducing antioxidant capacity
DPPH: 2,2-Diphenyl-1-picrylhydrazyl
DMEM-F12: Dulbecco's modified eagle medium/nutrient mixture F-12
DMSO: Dimethyl sulfoxide
DU-145: Human prostate carcinoma cell line
EDTA: Ethylenediaminetetraacetic acid
EDTAE: Ethylenediaminetetraacetic acid equivalents
EGFR: Epidermal growth factor receptor
FRAP: Ferric reducing antioxidant power
GAE: Gallic acid equivalents
GALAE: Galantamine equivalents
GO: Gene ontology
HCT-116: Human colorectal carcinoma cell line
HEK-293: Human embryonic kidney cells
HeLa: Human cervical cancer cell line
HT1080: Human fibrosarcoma cell line
KAE: Kojic acid equivalents
KEGG: Kyoto encyclopedia of genes and genomes
MCA: Metal chelating activity
MF: Molecular function
MTT: 3-(4,5-Dimethylthiazol-2-yl)-2,5-diphenyltetrazolium bromide
OMIM: Online mendelian inheritance in man
PBD: Phosphomolybdenum total antioxidant capacity assay
PBS: Phosphate-buffered saline
PPI: Protein–protein interaction
RE: Rutin equivalents
RNS: Reactive nitrogen species
ROS: Reactive oxygen species
RPMI-1640: Roswell park memorial institute 1640 medium
SH-SY5Y: Human neuroblastoma cell line
STAT3: Signal transducer and activator of transcription 3
TE: Trolox equivalents
TFC: Total flavonoid content
TNF: Tumor necrosis factor
TPC: Total phenolic content
UPLC-ESI-MS/MS: Ultra-performance liquid chromatography-electrospray ionization-tandem mass spectrometry

## Data Availability

Data will be made available from the authors on request.
